# Clinicopathological and Prognostic Characteristics of Malaysian Triple Negative Breast Cancer Patients Undergoing TAC Chemotherapy Regimen

**DOI:** 10.1155/2020/8424365

**Published:** 2020-04-01

**Authors:** Ahmad Aizat Abdul Aziz, Md Salzihan Md Salleh, Ravindran Ankathil

**Affiliations:** ^1^Human Genome Centre, School of Medical Sciences, Universiti Sains Malaysia, Health Campus, 16150 Kubang Kerian, Kelantan, Malaysia; ^2^Department of Pathology, School of Medical Sciences, Universiti Sains Malaysia, Health Campus, 16150 Kubang Kerian, Kelantan, Malaysia

## Abstract

Triple negative breast cancer (TNBC) is associated with aggressive tumour phenotype and early tumour relapse following diagnosis. Generally, clinicopathological features such as tumour size, patient's age at diagnosis, tumour histology subtypes, grade and stage, involvement of lymph nodes, and menopausal status are commonly used for predicting disease progression, prospects of recurrence, and treatment response. Prognostic value of clinicopathological features on Malaysian TNBC patients is limited. Thus, this study is aimed at investigating the association of clinicopathological features on disease-free survival (DFS) and overall survival (OS) of Malaysian TNBC patients undergoing TAC chemotherapy. Seventy-six (76) immunohistochemistry-confirmed TNBC patients were recruited. The clinicopathological features of TNBC patients were collected and recorded. Kaplan-Meier and log-rank followed by a Cox proportional hazard regression model were performed to evaluate the TNBC patients' survival. Out of 76 TNBC patients, 25 were chemoresistant and 51 were chemoresponders to the TAC chemotherapy regimen. The overall 5-year cumulative DFS and OS of TNBC patients were 63.5% and 76.3%, respectively. Multivariate Cox analysis demonstrated that medullary and metaplastic histology subtypes and positive axillary lymph node metastasis were significant prognostic factors associated with relapse with adjusted HR: 5.76, 95% CI: 2.35, 14.08 and adjusted HR: 3.55, 95% CI: 1.44, 8.74, respectively. Moreover, TNBC patients with medullary and metaplastic histology subtypes and positive axillary lymph node metastases had a higher risk to death than patients who had infiltrating ductal carcinoma and negative axillary lymph node metastasis (adjusted HR: 8.30, 95% CI: 2.38, 28.96 and adjusted HR: 6.12, 95% CI: 1.32, 28.42, respectively). Our results demonstrate the potential use of medullary and metaplastic histology subtype and positive axillary lymph node metastasis as a potential biomarker in predicting relapse and survival of the TNBC patients. This warrants further studies on intensification of chemotherapy and also identification and development of targeted therapy to reduce relapses and improve survival of TNBC patients.

## 1. Introduction

Breast cancer is a common malignant tumour that threatens a significant number of women worldwide. Triple negative breast cancer (TNBC) is one of the breast cancer subtypes characterized by negative immunohistochemistry (IHC) staining for estrogen (ER) and progesterone (PR) hormone receptors and no amplification of human epidermal growth receptor 2 (HER2) [1]. Worldwide, the incidence of TNBC represents approximately 10-24% of all breast cancer cases, and it is more common in Asian countries [2]. In Malaysia, the incidence of TNBC has been reported to range from 12.3% to 17.6% of the total breast cancer cases [3, 4].

TNBC is typically associated with high histological grade and stage as well as aggressive tumour phenotype showing only partial response to chemotherapy with lack of clinically established therapies [5]. Moreover, TNBC is strongly associated with distant recurrence, visceral metastases, and death when compared to other breast cancer types [6, 7]. To date, there are no approved targeted treatment available for TNBC patients other than chemotherapy. Although TNBC is chemosensitive, its treatment continues to be a challenge, due to tumour heterogeneity in TNBC histology subtypes [8]. Disease recurrence often occurs within the first 3 years of diagnosis, while the 5-year mortality rate appears to be increased following initial diagnosis [9]. Thus, a significant number of TNBC patients fail to respond or acquire resistance to the introduced chemotherapeutic agents that usually leads to a relapse and worsening of prognosis.

In predicting the disease progression, prospect of recurrence, and treatment response, clinicopathological features such as tumour size, patient's age at diagnosis, and involvement of lymph nodes are commonly used [10]. Thus, the present study was performed to analyse the clinicopathological features of TNBC patients undergoing taxane (docetaxel), adriamycin (doxorubicin), and cyclophosphamide (TAC) chemotherapy regimen and associate with their treatment outcome. It was presumed that the results from this study may provide information for the development of better management strategies for TNBC patients undergoing adjuvant TAC chemotherapy regimen.

## 2. Materials and Methods

### 2.1. Study Subjects

The study was approved by the Research Review Board and Ethics Committee of Universiti Sains Malaysia (USMKK/PPP/JEPeM [260.39210]) and Ministry of Health, Malaysia (NMRR-15-1200-25230), which complies with the Declaration of Helsinki. The study subjects were recruited from Hospital Universiti Sains Malaysia and Hospital Raja Perempuan Zainab II, Kota Bharu, Kelantan, Malaysia, while analysis was carried out at the Human Genome Centre, Universiti Sains Malaysia, Kubang Kerian, Kelantan.

Breast cancer patients who were histopathologically confirmed as negative ER, PR hormone receptors by immunohistochemistry (IHC) and no amplification of *HER 2*, who had undergone surgical resection, and who have completed six cycles of chemotherapy with TAC regimen was included in the study. Clinicopathological data of the patients such as age at diagnosis, tumour histology subtype, tumour stage, tumour histograde, status of lymph node metastasis, and menopausal status were recorded.

### 2.2. Evaluation of Treatment Response

Those TNBC patients who had completed six cycles of chemotherapy with TAC regimen were evaluated after one year. Those patients who developed disease progression, local recurrence, and primary and secondary tumours at different locations were categorized into the resistant group. On the other hand, TNBC patients who did not show any signs above were categorized into treatment response group. The treatment response was evaluated based on ultrasound, computed tomography (CT scan), or magnetic resonance imaging (MRI) findings by the treating oncologist.

### 2.3. Statistical Analysis

The cumulative disease-free survival (DFS) and overall survival (OS) analysis of TNBC patients were determined using Kaplan-Meier method and the log-rank test for single-factor analysis. Univariate and multivariate analyses were performed using Cox proportional hazard regression model. In the present study, the DFS was calculated as the time from first chemotherapy to the first locoregional or distant recurrence while OS was calculated as time from diagnosis to death or patient's last contact [11].

## 3. Results

A total of 76 histopathologically confirmed TNBC patients were recruited in this study. The mean age of the study subjects at diagnosis is 48.9 ± 9.67 years. The details of clinicopathological parameters, treatment status, and disease outcomes of the study subjects are shown in Table 1.

**Figure 1:**
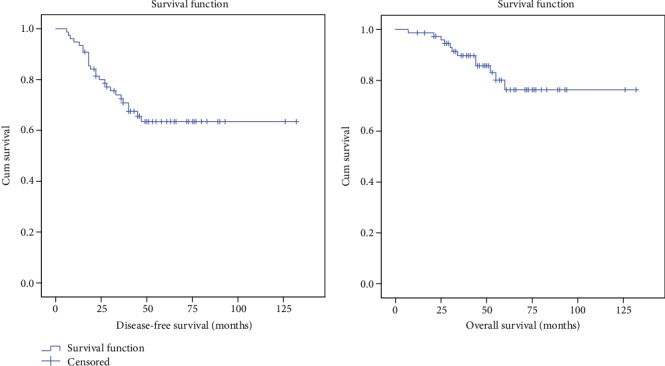
Cumulative disease-free survival and overall survival of TNBC patients.

The cohort study involved 76 TNBC patients for disease-free survival (DFS) and overall survival (OS) analyses with a median follow-up of 42.0 months (range 6-132 months) and 49.0 months (range 7-132 months), respectively. Out of 76 TNBC patients, 25 (33.0%) had relapsed over the mean follow-up period of 44.82 months. However, the overall median DFS and OS time of TNBC could not be determined due to incomplete information on follow-up for certain patients. The overall 5-year cumulative DFS and OS of TNBC patients were 63.5% and 76.3%, respectively (Figure 1).

**Figure 2:**
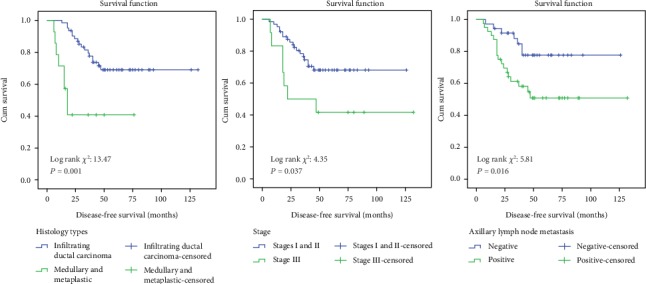
Kaplan-Meier curve of disease-free survival probability of TNBC patients according to histology subtype, stage, and axillary lymph node metastasis.

**Figure 3:**
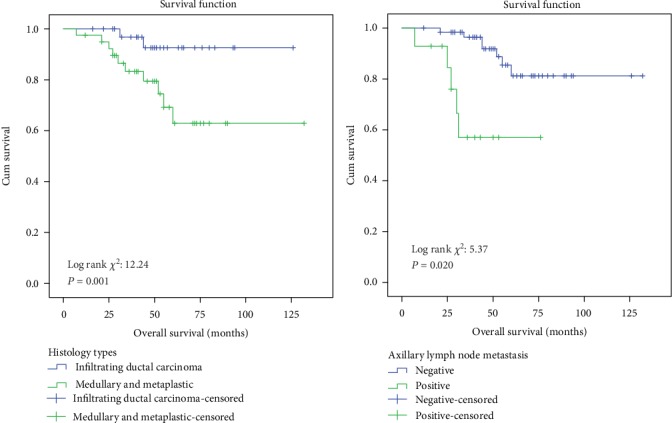
Kaplan-Meier curve of overall survival probability of TNBC patients according to histology subtype and axillary lymph node metastasis.

Significant differences were observed in DFS of TNBC patients with regard to the histology subtype, tumour stage, and axillary lymph node status metastasis (Figure 2). TNBC patients who had infiltrating ductal carcinoma subtype had a higher probability (69.0%) of DFS rates as compared to TNBC patients who had medullary and metaplastic subtypes (40.8%). Meanwhile, TNBC patients with stages I and II (68.1%) and negative axillary lymph node metastasis (77.7%) had a higher probability of DFS as compared to TNBC patients with stage III (41.7%) and positive axillary lymph node metastasis (50.9%), respectively. Meanwhile, factors such as the age group, tumour histograde, and menopausal status did not show any association with regard to DFS rates. With regard to the OS analysis, significant associations were observed in OS of TNBC patients with regard to the histology subtype and axillary lymph node metastasis (Figure 3). TNBC patients who had infiltrating ductal carcinoma subtype and negative axillary lymph node metastasis had significantly higher survival probability rates (81.2% and 92.6%, respectively) as compared to TNBC patients who had medullary and metaplastic subtypes (57.0%) and positive axillary lymph node metastasis (62.9%). However, factors such as the age group, tumour histograde, tumour stage, and menopausal status did not show any significant association with regard to overall survival rates.

A cox proportional hazard model for univariate Cox regression analysis was performed to determine the potential prognostic factors of DFS and OS in these 76 TNBC patients. TNBC patients who had medullary and metaplastic histology subtypes, and positive axillary lymph node metastasis had a significantly higher risk for relapse with a hazard ratio (HR) of 4.32, and 2.78, respectively. Moreover, TNBC patients who had medullary and metaplastic histology subtypes and positive axillary lymph node metastasis had a significant higher risk of death than patients with infiltrating ductal carcinoma and negative axillary lymph node metastasis (HR: 6.46, 95% CI: 1.97, 21.24 and HR: 5.01, 95% CI: 1.09, 22.90, respectively).

Accordingly, clinicopathological variables such as histology subtypes and axillary lymph node metastasis status were included and tested in a multivariate Cox regression model. From the present study, medullary and metaplastic subtypes and positive axillary lymph node metastasis emerged as significant prognostic factors for relapse after adjusting with other variables with adjusted HR: 5.76, 95% CI: 2.35, 14.08 and adjusted HR: 3.55, 95% CI: 1.44, 8.74, respectively. Moreover, TNBC patients with medullary and metaplastic histology subtypes had an approximately 8 times higher risk to death than patients who had infiltrating ductal carcinoma (HR: 8.30, 95% CI: 2.38, 28.96). Similarly, TNBC patients who had positive axillary lymph node metastases also had a higher risk to death than TNBC patients who had negative axillary lymph node metastasis (HR: 6.12, 95% CI: 1.32, 28.42) (Table 2).

## 4. Discussion

Prognostic biomarkers for TNBC are clearly warranted to identify the TNBC patients at higher risk of relapse or metastasis and to decide the appropriate strategies for surveillance and management of the patients. In the present study, clinicopathological data such as tumour histology subtypes, tumour stage, tumour histology grade, involvement of lymph node metastasis, and menopausal status were evaluated. DFS and OS analyses of TNBC patients were determined and correlated with all the studied parameters with the hope of identifying putative prognostic factors for TNBC patients. This is the first study conducted on prognostic analysis in Malaysian TNBC patients.

In the present study, the mean age of TNBC patients at diagnosis was 48.9 ± 9.67 years. Similarly, two previous studies on TNBC patients in Malaysia showed that the mean age of TNBC patients at age at diagnosis was 48.0 and 53.3 years [3, 4]. A recent study among Indonesian TNBC patients also demonstrated that an early mean age at diagnosis was 51.62 years [12]. This is similar with few other studies from different population, where the median age at diagnosis was between 48 and 50 years old [13–15]. A study reported that the pathological type of TNBC is similar with non-TNBC where invasive ductal carcinoma in TNBC is more frequent than non-TNBC [15]. The findings in the present study is in agreement with previous reports which observed high frequency of invasive ductal carcinoma in TNBC patients [4, 15, 16]. However, a few other histology subtypes such as medullary carcinoma, metaplastic carcinoma, apocrine carcinoma, mixed lobular-ductal carcinoma, lobular carcinoma, and adenoid cystic carcinoma were also reported in patients with TNBC [1].

TNBC is associated with a higher histological grade, marked cellular pleomorphism, high mitotic rate, and high atypical tumour cells [17–19]. The present finding is in agreement with previous studies which showed a high histological grade in two-third of TNBC patients [3, 14, 20]. Moreover, TNBC patients had relatively larger tumours and positively higher rate of axillary lymph node metastasis [21]. This is similar to the study by Qiu et al. [15] in which the frequency of TNBC patients who had lymph node metastases was higher (64.6%) as compared to those who had no axillary lymph node metastases (35.4%).

TNBC patients are more frequently reported in premenopausal patients of African-American origin [22, 23]. This was also similar to a report on Asian population, where majority of the TNBC patients were premenopausal [15]. In the present study, the frequency of TNBC patients with premenopausal status was higher as compared to postmenopausal status which is in concordance with previous reports [15, 20] and discordance with the study conducted by Dogra et al. [14].

TNBC is highly associated with a higher risk of recurrence and metastasis. From the 76 TNBC patients recruited, 33.0% did not respond to treatment (developed recurrence and metastasis). A study by Pogoda et al. [24] reported that 35.0% of TNBC patients developed recurrence in Polish population while 17.9% had treatment failure in the study by Dogra et al. (2014) in Indian population. In a Dutch cohort study, 16.0% of TNBC patients had regional, local, and distant recurrence [25]. A study reported by Qiu et al. showed that the frequency of recurrence or metastasis was higher in TNBC as compared to non-TNBC patients (27.95% vs. 13.38%, respectively) [15]. These findings which are in agreement with present study results indicated that TNBC patients have higher rate of recurrence or metastasis as compared to non-TNBC. Besides that, out of 25 TNBC patients who had relapse, 12 patients had already passed away during the present study duration.

Generally, TNBC patients have been reported to have a poorer prognosis as compared to non-TNBC [26]. In the present study, the cumulative 5 years of DFS and OS of TNBC patients were 63.5% and 76.5%, respectively. This is in agreement with several other studies that observed a higher DFS and OS in TNBC patients [21, 24, 26, 27]. Thus, it indicates that early-stage TNBC patients who underwent surgery and completed with cycles of TAC regimens had a higher DFS and OS. An earlier study demonstrated that the DFS rate of patients who received TAC was higher (75.0%) as compared to patients treated with FAC (68.0%) with a hazard ratio of 0.72 (*p* = 0.001). Moreover, the OS rate at five years was 87.0% for TAC and 81.0% for FAC, with a hazard ratio of 0.72 (*p* = 0.008) [28]. In another study, Martin et al. [29] demonstrated that TAC was superior to FAC (5-fluorouracil, doxorubicin, cyclophosphamide) regimen in treating TNBC. Recently, a retrospective study on TNBC patients treated with CAF (cyclophosphamide, doxorubicin, 5-fluorouracil), AC-T (doxorubicin, cyclophosphamide followed by docetaxel), and TAC regimens showed that treatment with TAC regimen resulted in an increased DFS and OS in TNBC patients [20].

The present study also has a few limitations. First, the present study is limited by a small number of TNBC patients which interfered the power of the study. This has been reflected in present results where *p* value was not significant despite high HR value and also where wide 95% confident interval (CI) and standard error (SE) ranges were observed. But the fact that TNBC is the rarest among all other breast cancer subtypes needs to be considered. In Malaysia, a previous study demonstrated that the frequency of TNBC ranged from 12.3% to 17.6% of the total breast cancer cases. Thus, recruiting an adequate number of patients who were clinically and histopathologically confirmed as TNBC has been a challenge for the present study. Additionally, selection of the TNBC patients who had undergone surgery and completed six cycles of adjuvant TAC chemotherapy as per the inclusion criteria was also a challenging task. Secondly, incomplete patient follow-up for at least 5 years after completion of six cycles of chemotherapy and loss on follow-up were also other limitations. Because of this reason, the median of survival time (in DFS and OS analysis), which is essential for survival analysis, could not be determined.

## 5. Conclusion

To the best of available knowledge, this is the first study to evaluate the association of clinicopathological parameters with TAC chemotherapy response in TNBC patients. It is reasonable to conclude that medullary and metaplastic histology subtypes and positive axillary lymph node metastasis could be useful as biomarkers in predicting TAC chemotherapy response in TNBC patients. These clinicoparameters could help clinicians and oncologists in early identification (before the chemotherapy initiation) of TNBC patients who might respond better with chemotherapy and who might end up with poor outcomes and recurrences.

## Figures and Tables

**Table 1:** Clinicopathological features of TNBC patients.

Characteristics		Frequency (*N* = 76)
Age	Mean age at diagnosis ± SD	48.9 ± 9.67

Type	Infiltrating ductal carcinoma (IDC)	63 (82.0%)
	Other (Medullary and Metaplastic)	14 (18.0%)

Histological grade	I and II	34 (45.0%)
	III	42 (55.0%)

Stage	I and II	64 (84.0%)
	III	12 (16.0%)

Lymph node status	Negative	36 (47.0%)
	Positive	40 (53.0%)

Menopausal status	Pre-menopausal	49 (65.0%)
	Post-menopausal	27 (35.0%)

Treatment status	Responder	51 (67.0%)
	Non-responder	25 (33.0%)

Status	Alive	64 (84.0%)
	Death	12 (16.0%)

**Table 2:** Univariate and multivariate Cox analyses on clinicopathological variables and survival of TNBC patients.

Clinicopathological variables	Disease-free survival				Overall survival			
	Crude HR^∗^	Adjusted HR^∗∗^	Wald statistics (df)	*p* value	Crude HR^∗^	Adjusted HR^∗∗^	Wald statistics (df)	*p* value
Histology subtype								
Infiltrating ductal carcinoma	1.00	1.00			1.00	1.00		
Other (medullary and metaplastic)	4.32 (1.83, 10.15)	5.76 (2.35, 14.08)	14.71 (1)	**0.001** ^∗∗∗^	6.46 (1.97, 21.24)	8.30 (2.38-28.96)	11.01 (1)	**0.001** ^∗∗∗^
Axillary lymph node metastasis								
Non-metastasis	1.00	1.00			1.00	1.00		
Metastasis	2.78 (1.16, 6.67)	3.55 (1.44, 8.74)	7.62 (1)	**0.006** ^∗∗∗^	5.01 (1.09, 22.90)	6.12 (1.32, 28.42)	5.43 (1)	**0.021** ^∗∗∗^

^∗^Univariate Cox regression; ^∗∗^multivariate Cox regression. ^∗∗∗^*p* value < 0.05, statistically significant based on multivariate Cox regression analysis.

## Data Availability

The data used to support the findings of this study are available from the corresponding author upon request.

## Abbreviations

AC-T: Doxorubicin, cyclophosphamide followed by docetaxel
CAF: Cyclophosphamide, doxorubicin, 5-fluorouracil
FAC: 5-Fluorouracil, doxorubicin, cyclophosphamide
TAC: Docetaxel, doxorubicin, cyclophosphamide.
